# YAP1 overexpression contributes to the development of enzalutamide resistance by induction of cancer stemness and lipid metabolism in prostate cancer

**DOI:** 10.1038/s41388-021-01718-4

**Published:** 2021-03-04

**Authors:** Hsiu-Chi Lee, Chien-Hui Ou, Yun-Chen Huang, Pei-Chi Hou, Chad J. Creighton, Yi-Syuan Lin, Che-Yuan Hu, Shih-Chieh Lin

**Affiliations:** 1 Department of Physiology, College of Medicine, National Cheng Kung University, Tainan, Taiwan; 2 Department of Urology, National Cheng Kung University Hospital, College of Medicine, National Cheng Kung University, Tainan, Taiwan; 3 Institute of Molecular Medicine, College of Medicine, National Cheng Kung University, Tainan, Taiwan; 4 Department of Medicine, Dan L. Duncan Cancer Center Division of Biostatistics, Baylor College of Medicine, Houston, TX USA; 5 Institute of Basic Medical Sciences, College of Medicine, National Cheng Kung University, Tainan, Taiwan; 6 Institute of Clinical Medicine, College of Medicine, National Cheng Kung University, Tainan, Taiwan

**Keywords:** Cancer therapeutic resistance, Transcription

## Abstract

Metastatic castration-resistant prostate cancer (mCRPC) is a malignant and lethal disease caused by relapse after androgen-deprivation (ADT) therapy. Since enzalutamide is innovated and approved by US FDA as a new treatment option for mCRPC patients, drug resistance for enzalutamide is a critical issue during clinical usage. Although several underlying mechanisms causing enzalutamide resistance were previously identified, most of them revealed that drug resistant cells are still highly addicted to androgen and AR functions. Due to the numerous physical functions of AR in men, innovated AR-independent therapy might alleviate enzalutamide resistance and prevent production of adverse side effects. Here, we have identified that yes-associated protein 1 (YAP1) is overexpressed in enzalutamide-resistant (EnzaR) cells. Furthermore, enzalutamide-induced YAP1 expression is mediated through the function of chicken ovalbumin upstream promoter transcription factor 2 (COUP-TFII) at the transcriptional and the post-transcriptional levels. Functional analyses reveal that YAP1 positively regulates numerous genes related to cancer stemness and lipid metabolism and interacts with COUP-TFII to form a transcriptional complex. More importantly, YAP1 inhibitor attenuates the growth and cancer stemness of EnzaR cells in vitro and in vivo. Finally, YAP1, COUP-TFII, and miR-21 are detected in the extracellular vesicles (EVs) isolated from EnzaR cells and sera of patients. In addition, treatment with EnzaR-EVs induces the abilities of cancer stemness, lipid metabolism and enzalutamide resistance in its parental cells. Taken together, these results suggest that YAP1 might be a crucial factor involved in the development of enzalutamide resistance and can be an alternative therapeutic target in prostate cancer.

## Introduction

Prostate cancer (PCa) is the most common cancer and a leading cause of cancer death in men worldwide [1]. When men are diagnosed with local PCa, they will be treated with therapies such as radical prostatectomy or radiotherapy [2]. Most of them can be efficiently cured after those therapies. However, some patients with local advanced or metastatic PCa will receive androgen-deprivation therapy (ADT) through useage of gonadotropin-releasing hormone agonizts and a combination of AR antagonists such as biculatamide to block androgen function since androgen is a crucial factor for the PCa development [3]. Although ADT therapy is initially effective for advanced PCa, nearly all metastatic prostate tumors will still relapse within a period of 2–3 years despite androgen suppression, a stage called metastatic castration-resistant PCa (mCRPC). Unfortunately, treatment option for mCRPC is limited before 2010. Only docetaxel chemotherapy has shown the 2–3 months prolongation of survival benefit for mCRPC patient at 2004 [4, 5], resulting in approved therapeutic option by US Food and Drug Administration (FDA) and as first line therapy worldwide. However, nearly all mCRPC patients treated with docetaxel eventually develop drug resistance for this chemoreagent and accompanied with high mortality rate. Thus, to innovate effective therapy for mCRPC is a critical issue for PCa.

Recently, enzalutamide is one of several second-generation antiandrogen drugs for mCRPC patient in the post-docetaxel setting due to its survival benefit [6, 7]. Later, indication for enzalutamide is extended for inclusion of chemotherapy-naive mCRPC patients [8] and even for nonmetastatic CRPC patients in 2018 [9]. Although enzalutamide exploit the treatment option for mCRPC patient, drug resistance for enzalutamide inevitably occurs. Several studies have revealed that underlying mechanisms responsible for enzalutamide resistance. For examples, specific missense mutation of AR F876L caused by enzalutamide contributes to its resistance [10]. Furthermore, increases of androgen receptor splice variant 7 (AR-V7) transcript and glucocorticoid receptor (GR) are the underlying mechanisms contributing to enzalutamide resistance [11, 12]. However, most of the aforementioned findings only focus on androgen-AR function causing drug resistance and several side effects are reported during ADT therapy due to critical functions of AR in the normal prostate [13]. Therefore, it is important to develop an effective androgen-independent therapy for mCRPC patient with enzalutamide resistance.

Yes-Associated Protein 1 (YAP1) is a critical transcriptional regulator negatively suppressed by core kinase cascade of Hippo tumor suppressor pathway [14]. When Hippo pathway is activated, the upstream kinase, LATS1/2, which is activated by MST1/2, will phosphorylate YAP1 and repress its transcriptional activity by promoting YAP1 accumulation in the cytoplasm and degradation via ubiquitin-mediated proteolysis [15, 16]. In contrast, inactivation of LATS1/2 kinase activity will promote nuclear translocation of YAP1 and interact with several crucial transcriptional factors (TFs) such as TEAD and SMAD families to regulate downstream gene expression [17]. Several studies have revealed the critical roles of YAP1 in the PCa development. Nguyen et al. have found that YAP1 overexpression in the mouse prostate epithelium can promote age-dependent PCa development [18]. Furthermore, YAP1 expression is further increased in the CRPC cells and its higher level is critical for the CRPC growth and invasion in vitro and in vivo [19, 20]. Clinically, YAP1 is indeed overexpressed in the mCRPC specimens [19]. Interestingly, Kuser-Abali et al. have demonstrated that YAP1 activation by MST1 knockdown in androgen sensitive LNCaP cells can promote its androgen-independent growth [21]. Furthermore, YAP1 and AR consistently interact with each other in the androgen-independent PCa cells [21]. These results suggest that the interaction between YAP1 and AR contributes to the switch from androgen-dependent to castration-resistant growth in PCa. Taken together, although YAP1 has been revealed to play crucial roles in PCa, the role of YAP1 in resistance to second-generation antiandrogen drug is still unexamined.

Herein, we have shown that YAP1 overexpression contributes to the development of enzalutamide resistance via increasing abilities of cancer stemness and lipid metabolism. More importantly, YAP1 has therapeutic and diagnostic potentials for enzalutamide resistance.

## Result

### YAP1 is a crucial factor involved in the development of enzalutamide resistance in PCa

**Fig. 1:**
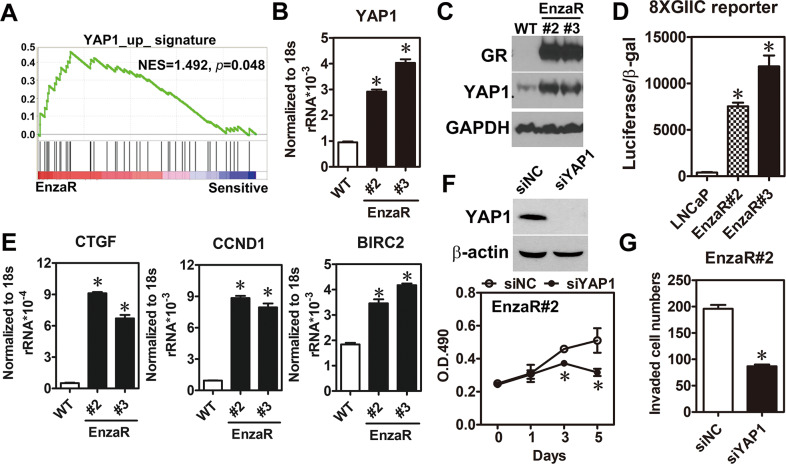
Overexpression of YAP1 was critical for growth and invasion of enzalutamide-resistant cells. **A** YAP1 signature was obtained from molecular signatures database of GSEA web site (http://software.broadinstitute.org/gsea/msigdb/index.jsp) and cross-referenced with the enzalutamide-resistant dataset (GSE52169). **B**, **C** YAP1 mRNA and protein were measured in parental LNCaP cells (WT) and enzalutamide-resistant cells (EnzaR) by RT-qPCR (*n* = 3) and western blot, respectively. Glucocorticoid receptor (GR) was used as positive marker for EnzaR cells. Asterisk indicates *p* < 0.05 using one-way ANOVA test. **D** YAP1 transcriptional activity was measured by transient transfection of 8xGIIC luciferase reporter into parental LNCaP cells (WT) and enzalutamide-resistant cells (EnzaR) (*n* = 3). Asterisk indicates *p* < 0.05 using one-way ANOVA test. **E** Several well-known YAP1 targets were assayed by RT-qPCR in parental LNCaP cells (WT) and enzalutamide-resistant cells (EnzaR) (*n* = 3). Asterisk indicates *p* < 0.05 using one-way ANOVA test. **F** YAP1 was knocked down in EnzaR cells by siRNA against YAP1 (40 nM). Results of western blot (upper panel) showed the knockdown efficiency of siYAP1 and cell proliferation (lower panel) was measured by MTS assay for indicated time point (*n* = 3). Asterisk indicates *p* < 0.05 using two-way ANOVA test. **G** Enzalutamide-resistant cells knocked down YAP1 expression by siRNA for 48 h were trypsinized and performed cell invasion assay for additional 16 h. Average of invaded cell numbers were counted from six different areas. Asterisk indicates *p* < 0.05 using two-tailed Student’s *t*-test.

To identify the dysregulation of signaling pathway involving in the development of enzalutamide resistance, signatures of the enzalutamide-resistant (EnzaR) tumor expression profiles from the public dataset (GSE52169) were analyzed by using gene set enrichment analysis (GSEA). YAP1 signature was enriched in the phenotype of enzalutamide resistance (Fig. 1A) and a similar result was observed in another EnzaR dataset (GSE51873) (Supplementary Table 1). Next, YAP1 expression in EnzaR cells set up from our previous study [22] was investigated to validate the findings of bioinformatics analyses. Results showed that YAP1 expression (Fig. 1B, C), transcriptional activity (Fig. 1D), and its downstream target genes (Fig. 1E) were markedly increased in the EnzaR cells compared to its LNCaP parental cells. Furthermore, YAP1 knockdown retarded the growth and invasion of EnzaR cells. (Fig. 1F, G). These results reveal that YAP1 plays crucial roles in the development of enzalutamide resistance.

### YAP1 overexpression in EnzaR cells is mediated by androgen-deprivation-induced COUP-TFII expression

Due to YAP1 expression was elevated in the EnzaR cells, we tried to investigate its underlying mechanism. Since enzalutamide directly inhibits the function of androgen receptor (AR), we hypothesize that YAP1 expression might be regulated by androgen-AR axis. To test the idea, LNCaP cells were cultured under androgen-deprivation treatment. As shown in Supplementary Fig. 1A, androgen-deprivation significantly increased YAP1 expression while adding synthetic androgen compound, R1881, reduced its expression. Similar result showed that YAP1 expression was elevated in the androgen-independent cell line, Abl (Supplementary Fig. 1B). Furthermore, direct knockdown of AR in LNCaP cells stimulated YAP1 expression under normal culture condition (Supplementary Fig. 1C). These findings suggest that YAP1 was AR-repressed target gene.

**Fig. 2:**
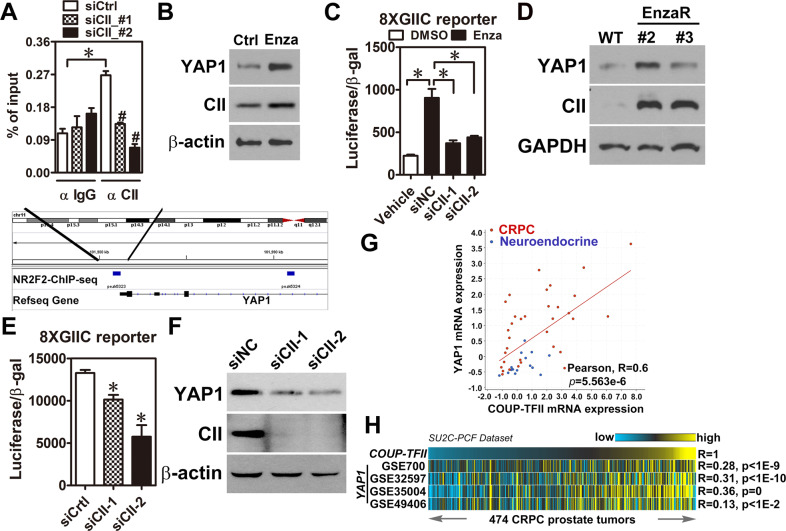
COUP-TFII positively and directly regulates YAP1 expression at the transcriptional level in EnzaR cells. **A** EnzaR (clone#2) cells were transiently transfected with siRNA against COUP-TFII (CII) for 48 h and performed COUP-TFII-chromatin immunoprecipitation (ChIP). Primers were designed according to COUP-TFII-ChIP-seq analysis result and used for ChIP-qPCR (*n* = 3). Blue rectangle indicated COUP-TFII binding peak located in the YAP1 gene locus (lower panel). **B** YAP1 and COUP-TFII (CII) expression levels in LNCaP cells treated with 10 μM Enzalutamide for 72 h. **C** YAP1 transcriptional reporter (8XGIIC) and siRNA against COUP-TFII or control were simultaneously transfected into LNCaP cells treated with or without 10 μM Enzalutamide for 72 h. Cells were lysed and luciferase activity was measured (*n* = 3). Asterisk indicates *p* < 0.05 using two-tailed Student’s *t*-test. **D** YAP1 and COUP-TFII expression levels in LNCaP cell (WT) and two different enzalutamide-resistant clones (#2 and #3). **E** EnzaR (clone#2) cells were transiently transfected with siRNA against COUP-TFII and YAP1 transcriptional reporter (8XGIIC) for 48 h. Cells were lysed and measured luciferase activity (*n* = 3). Asterisk indicates *p* < 0.05 using one-way ANOVA test. **F** COUP-TFII was knocked down by two different siRNA (40 nM) for 48 h. YAP1 and COUP-TFII expression levels were determined by western blot. β-actin was detected as a loading control. **G** YAP1 and COUP-TFII expression levels were performed Pearson correlation analysis by using a neuroendocrine PCa dataset (*n* = 49) deposited in the cBioportal (https://www.cbioportal.org/). **H** Gene signatures of COUP-TFII and YAP1 from four different datasets were analyzed their signature correlations by Pearson correlation analyses in a large cohort of mCRPC dataset (*n* = 474) downloaded from cBioPortal. Yellow color, high-signature scoring in prostate tumor specimens, represents high manifestation of associated transcriptional patterns while blue color, equals low-signature scoring. Correlation between COUP-TFII signature and YAP1 signatures were tested by Pearson’s correlation.

Next, a public dataset performing AR-ChIP-seq in LNCaP cells treated with vehicle or R1881 (GSE45124) was analyzed to identify the potential AR binding site located in the YAP1 locus. Although AR had a physical binding site near transcriptional starting site in the YAP1 locus, AR binding signals were not different between vehicle and R1881 treatment (Supplementary Fig. 1D), suggesting that AR may indirectly repress YAP1 expression. Previously, we have found that COUP-TFII expression was increased in the EnzaR cells [22] and its expression was directly repressed by AR (Supplementary Fig. 1A–C). Interestingly, re-analysis of COUP-TFII-ChIP-seq from another public dataset (GSE52008) revealed a COUP-TFII binding site in the promoter region of YAP1 locus (Fig. 2A, lower panel) and COUP-TFII indeed bound to the YAP1 locus in LNCaP cells (Fig. 2A, upper panel). Moreover, AR directly bound to COUP-TFII locus (Supplementary Fig. 1E, F). Therefore, we hypothesize that AR inhibition by enzalutamide might lead to YAP1 overexpression via COUP-TFII function. Our findings showed that YAP1 and COUP-TFII expression were simultaneously increased at the RNA and protein levels after enzalutamide treatment (Fig. 2B and Supplementary Fig. 2A). Then, LNCaP cells with or without COUP-TFII kncokdown were treated with or without enzalutamide to investigate its effect on YAP1 function. Results showed that enzalutamide-induced YAP1 transcriptional activity was abolished after COUP-TFII knockdown in LNCaP cells (Fig. 2C). In addition, both RNA and protein levels of YAP1 and COUP-TFII were highly increased in the EnzaR cells (Fig. 2D and Supplementary Fig. 2B). Furthermore, YAP1 level and transcriptional activity were decreased in EnzaR cells with COUP-TFII knockdown (Fig. 2E, F). More importantly, YAP1 expression was positively correlated with COUP-TFII expression in a PCa dataset containing CRPC and neuroendocrine specimens (Fig. 2G). Similarly, analyses of YAP1 signatures from four different resources and COUP-TFII signature revealed their significantly positive correlations in a large cohort of clinical mCRPC dataset (Fig. 2H), suggesting a clinical relevance of COUP-TFII-YAP1 regulation. Taken together, these results reveal that YAP1 overexpression is mediated by COUP-TFII function at the transcriptional level in the EnzaR cells.

### COUP-TFII-induced miR-21 expression is an indirect manner causing YAP1 overexpression in EnzaR cells

**Fig. 3:**
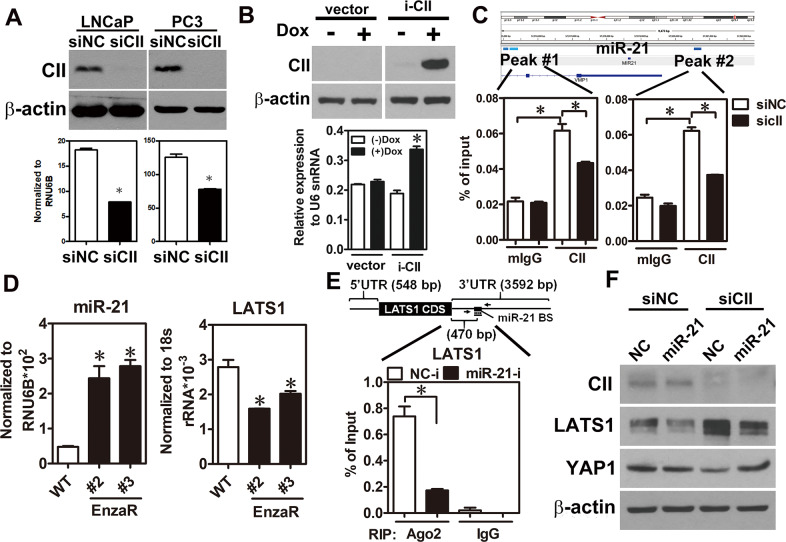
COUP-TFII-induced miR-21 expression causes LATS1 downregulation and leads to increase of YAP1 level in EnzaR cells. **A** LNCaP or PC3 cells were knocked down by control siRNA (siNC) or siRNA against COUP-TFII (siCII) for 48 h. Western blot results showed the knockdown efficiency of siRNA against COUP-TFII (upper panel). miR-21 and RNU6B expression levels were measured by realtime PCR (*n* = 3) and results were presented as miR-21 normalized to RNU6B (lower panel). Asterisk indicates *p* < 0.05 using two-tailed Student’s *t*-test. **B** Doxycycline (0.5 μg/ml) was used to treat RWPE-1 cells carrying with empty vector (vector) or inducible COUP-TFII construct (i-CII) for 48 h. Western blot result demonstrated the expression level of COUP-TFII after doxycycline treatment (upper panel). miR-21 and RNU6B expression levels were measured by realtime PCR (*n* = 3) and results were presented as miR-21 normalized to RNU6B (lower panel). Asterisk indicates *p* < 0.05 using two-tailed Student’s *t*-test. **C** LNCaP cells were used to perform COUP-TFII Chromatin- immunoprecipitation (ChIP)-PCR. Cartoon of miR-21 locus showed the two potential COUP-TFII binding regions. Specific primers were individually designed to amplify those regions. Results were presented as percentage of input control (*n* = 3). Asterisk indicates *p* < 0.05 using two-tailed Student’s *t*-test. **D** Expression levels of miR-21 and LATS1 were measured in parental LNCaP (WT) cells and enzalutamide-resistant (enzaR) clones by realtime PCR (*n* = 3). Asterisk indicates *p* < 0.05 using one-way ANOVA test following by Dunnett analysis. **E** EnzaR cells were treated miR-21 inhibitor (miR-21-i) for 48 h and then performed Ago2-RNA-immunoprecipitation (RIP). Specific primer was designed to amplify LATS1 3′UTR region containing miR-21-targeting site. Results were presented as percentage of input control. Asterisk indicates *p* < 0.05 using two-tailed Student’s *t*-test. **F** EnzaR cells were co-treated control mimic or miR-21 mimic (50 nM) and control siRNA or siRNA against COUP-TFII (40 nM) for 48 h. COUP-TFII (CII), YAP1, LATS1, and β-actin expression levels were detected by western blot.

Since COUP-TFII knockdown-decreased YAP1 expression was more obvious at the protein level compared to its RNA level reduced by COUP-TFII knockdown (Supplementary Fig. 3A and Fig. 2F), these findings suggest that an additional regulation at the post-transcriptional level might involve in COUP-TFII-induced YAP1 expression in EnzaR cells. ChIP-atlas and microRNA target analysis from microRNA.org revealed that COUP-TFII has a binding signal in the promoter region of miR-21 locus and LATS1, a negative regulator of YAP1, is the potential target of miR-21 (Fig. 3C, upper panel and Supplementary Fig. 3B), suggesting that COUP-TFII may indirectly regulate YAP1 level via miR-21-mediated LATS1 expression. Next, miR-21 expression level was determined in COUP-TFII loss-of-function or gain-of-function cells, respectively. Results showed that miR-21 expression was decreased in COUP-TFII-knocked down LNCaP and PC3 cells while increased in COUP-TFII-overexpressed RWPE-1, a normal prostate epithelial cells (Fig. 3A, B). Furthermore, ChIP-qPCR revealed that COUP-TFII specifically bound to the promoter region of miR-21 locus in LNCaP cells (Fig. 3C, lower panel). Importantly, miR-21 level was elevated while LATS1 level was decreased in the EnzaR cells compared to its parental cells (Fig. 3D). Furthermore, results of Ago2-RNA-IP also demonstrated that miR-21 physically bound to 3′UTR region of LATS1 mRNA and it was abolished when miR-21 inhibitor was treated in EnzaR cells (Fig. 3E). Finally, our results revealed that COUP-TFII knockdown increased LATS1 expression while decreased YAP1 expression, and these phenomena were reversed after restore of miR-21 expression in EnzaR cells (Fig. 3F). Taken together, these findings suggest that COUP-TFII can indirectly increase YAP1 expression via miR-21-LATS1 regulation axis.

### YAP1 contributes to enzalutamide resistance via positive regulation of cancer stemness and lipid metabolism

**Fig. 4:**
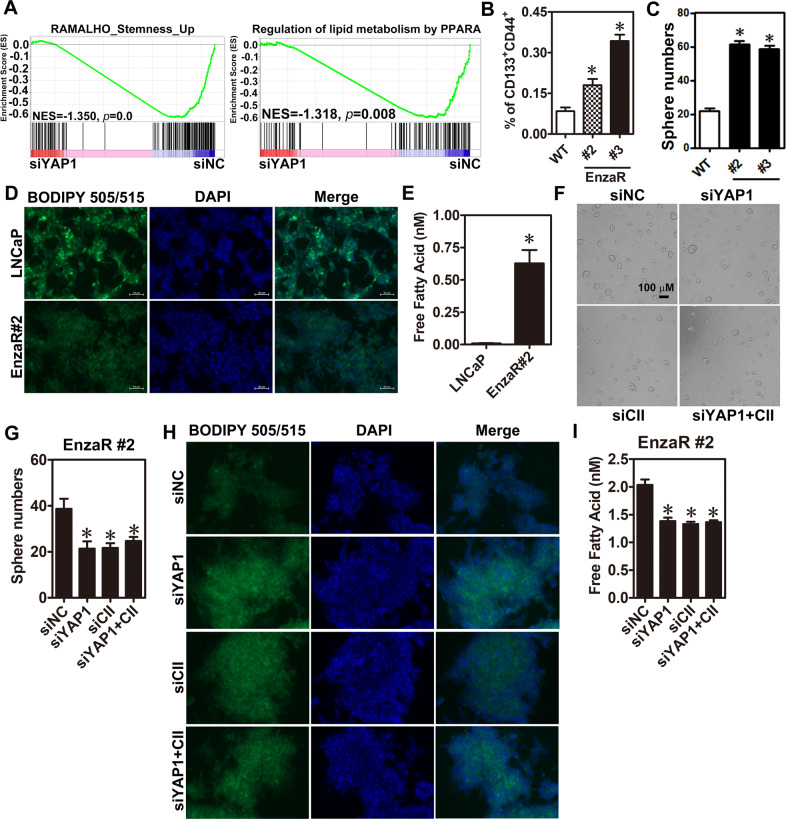
Increase of YAP1 expression promotes cancer stemness and lipid metabolism in EnzaR cells. **A** RNA-seq results of YAP1 knockdown and its control in EnzaR cells were performed GSEA analyses. **B** Enzalutamide-resistant (EnzaR#2 and #3) and its parental cells (WT) were analyzed by flow cytometry. CD133 and CD44 double-positive cells were cells with cancer stemness (*n* = 3). **C** Enzalutamide-resistant (EnzaR#2 and #3) and its parental cells (WT) were performed sphere culture for 1 week. Results were presented as sphere number (*n* = 3). EnzaR#2 and its parental LNCaP cells were performed lipid staining by BODIPY 505/515 dye (**D**) and measured free fatty acid level by a commercial kit (**E**). **E** COUP-TFII (CII), YAP1, and BMI1 expression levels were detected by RT-qPCR in CD133^−^ and CD133^+^ cells with stable knockdown of COUP-TFII or control construct sorted by CD133 magnetic beads. Results were normalized to 18s rRNA expression. **F**, **G** EnzaR#2 cells were treated with control (siNC), YAP1, or CII siRNA for 48 h. Then, those cells were trypsinized and performed sphere culture for 7 days. Representative pictures (**F**) and quantifications of sphere numbers (**G**) were shown. EnzaR#2 cells were individually or simultaneously knocked down YAP1 or COUP-TFII in EnzaR cells for 48 h and performed lipid staining (**H**) and measured free fatty acid level (**I**). Asterisk indicates *p* < 0.05 using one-way ANOVA test following by Dunnett analysis (**B, C, G, I**) and two-tailed Student’s *t*-test (**E**).

To investigate the function of YAP1 in developing enzalutamide resistance, EnzaR cells with or without YAP1 knockdown were performed RNA-seq analysis. GSEA analyses revealed that signatures of cancer stemness and lipid metabolism, a process related to cancer stemness [23], development of CRPC and enzalutamide resistance [24, 25], were enriched in the phenotype of knockdown control (Fig. 4A). Vise versa, analyses of YAP1 and COUP-TFII signatures were shown to enrich in the PCa stem cell (PCSC) phenotypes by using GSEA analysis (Supplementary Fig. 4A). Furthermore, metascape analysis for downregulation gene list of YAP1 knockdown in EnzaR cells revealed that several biological processes were related with lipid metabolism (Supplementary Fig. 4B). These findings suggest that YAP1 might regulate cancer stemness and lipid metabolism to promote enzalutamide resistance. First, to test the roles of COUP-TFII and YAP1 in cancer stemness, sphere culture and magnetic activated cell sorting (MACS) were used to enrich PCSC population by using androgen insensitive cells (DU145 and PC3). Results showed that COUP-TFII, YAP1, and other stemness-related gene expression levels were markedly elevated in the sphere culture condition (Supplementary Fig. 4C). Similar results were observed in the CD133^+^ cells sorted by MACS (Supplementary Fig. 4D). Furthermore, knockdown of COUP-TFII not only reduced expression levels of YAP1 and stemness-related genes but also decreased sphere numbers in PC3 cells (Supplementary Fig. 4D, E). These findings suggest both COUP-TFII and YAP1 may promote cancer stemness.

Next, LNCaP and EnzaR cells from two different clones were detected PCSC markers (CD133^+^ and CD44^+^) by using flow cytometry and performed sphere culture. Results showed that EnzaR cells indeed had higher PCSC population (Fig. 4B), sphere number (Fig. 4C) and stemness-related gene expression (Supplementary Fig. 4F). Furthermore, lipid content was obviously reduced while free fatty acid level was markedly increased in EnzaR cells (Fig. 4D, E). Then, COUP-TFII and YAP1 were respectively knocked down by their siRNAs to investigate their roles in enzalutamide-promoted PCSC population and lipid metabolism. Results showed that individual and simultaneous knockdown of COUP-TFII and YAP1 decreased sphere formation (Fig. 4F, G), restored lipid content (Fig. 4H) and reduced free fatty acid level (Fig. 4I) in EnzaR cells. Taken together, these results reveal that enzalutamide-increased COUP-TFII and YAP1 expression levels contribute to enriching PCSC population and promoting alternation of lipid metabolism.

### COUP-TFII and YAP1 co-regulate downstream target genes related to cancer stemness

**Fig. 5:**
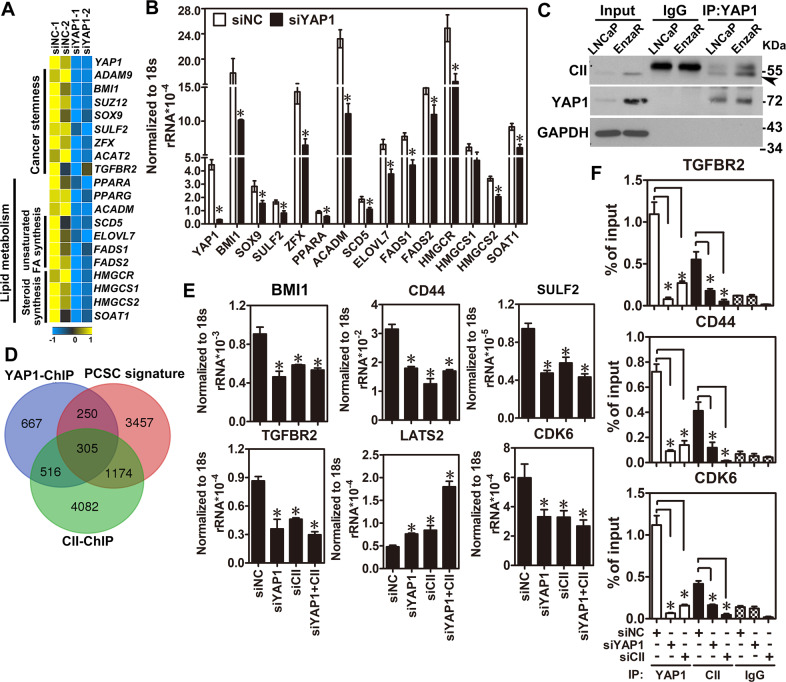
Interaction between COUP-TFII and YAP1 co-regulate cancer stemness-related gene expression in EnzaR cells. **A** EnzaR cells were treated with siRNA against YAP1 or control siRNA for 48h. Next, YAP1 downstream gene expression profile and potential function of YAP1 knockdown was analyzed by RNA-seq and GSEA analyses. **B** Genes related to cancer stemness and lipid metabolism were verified by qRT-PCR after YAP1 knockdown in EnzaR cells for 48h (*n* = 3). *p* < 0.05 using two-tailed Student’s *t*-test. **C** YAP1 antibody was used to pull down endogenous YAP1 protein and measured COUP-TFII expression in both LNCaP and EnzaR cells. **D** Gene lists from YAP1-ChIP-seq (GSE66081) and COUP-TFII-ChIP-seq (GSE52008) were cross-referenced with PCa stem cell signature (PCSC) derived from public dataset (GSE19713) to identify COUP-TFII and YAP1 co-regulated downstream targets that paly roles in cancer stemness. **E** The expression levels of COUP-TFII and YAP1 were individually or simultaneously knocked down siRNA against COUP-TFII or YAP1 (40 nM) for 48h in EnzaR cells. COUP-TFII, YAP1, and several expression levels of genes related to cancer stemness were determined by RT-qPCR. Results were normalized to 18s rRNA expression (*n* = 3). Asterisk indicates *p* < 0.05 using one-way ANOVA test following by Dunnett analysis. **F** EnzaR cells were individually knocked down COUP-TFII and YAP1 by siRNAs for 48 h. Next, ChIP quantitative PCR was performed in those cells by using COUP-TFII and YAP1 antibody. Results were presented as percentage of input control. Asterisk indicates *p* < 0.05 using one-way ANOVA test for above experiments.

Since both functions of YAP1 and COUP-TFII enriched PCSC population and promoted lipid metabolism, we thus tried to investigate its underlying mechanism. First, genes from signatures of cancer stemness and lipid metabolism were checked from results of GSEA analyses (Fig.4A) and reorganized some of them to present as heatmap shown in Fig. 5A. Next, those genes were verified by qRT-PCR in EnzaR cells with YAP1 knockdown. Results showed that most of their expression levels were decreased after knockdown of YAP1 in EnzaR cells (Fig. 5B). Since COUP-TFII and YAP1 are transcriptional regulators and their expression levels are elevated in the EnzaR cells, it is reasonable to suspect that both of them might form a complex to regulate downstream target gene expression. To test the idea, both COUP-TFII-FLAG and YAP1-GFP constructs were transfected into 293FT cells and performed immunoprecipitation by using FLAG antibody to pull down exogenous COUP-TFII and detect YAP1 expression. Results showed that COUP-TFII interacted with YAP1 in 293FT cells (Supplementary Fig. 5A). Similar results were observed when LNCaP and EnzaR cells were used to pull down endogenous COUP-TFII or YAP1. Interestingly, the interaction between COUP-TFII and YAP1 was more obvious in EnzaR cells (Fig. 5C and Supplementary Fig. 5B). Next, three public datasets performed COUP-TFII-ChIP (GSE52008), YAP1-ChIP (GSE61852) and PCSC gene expression signature (GSE19713), and lipid metabolism-related gene list downloaded from GeneOntology were re-analyzed and cross-referenced with those results to investigate whether COUP-TFII/YAP1 complex could co-regulate genes related to cancer stemness or lipid metabolism. Surprisingly, 305 genes were contained both COUP-TFII and YAP1 binding signals in gene loci related to PCSC but none of them was identified in gene loci related to lipid metabolism (Fig. 5D and Supplementary Fig. 5C), suggesting COUP-TFII/YAP1 complex might directly co-regulate cancer stemness genes. To further analyze the functions of those 305 genes, they were involved in the cell differentiation, cell cycle regulation, apoptosis, angiogenesis, androgen signaling, and stemness (Supplementary Table 2) after metacore, an integrated software suite for functional analysis. As a proof-of-concept, several genes such as TGFBR2, CDK6, CD44, BMI1, LATS2, and SULF2 were chosen from the corresponding processes to validate the findings of bioinformatics analyses. Results showed that knockdown of COUP-TFII or YAP1 decreased CD44, BMI1, and SULF2 while increased LATS2 expression levels (Fig. 5E and Supplementary Fig. 5D). Next, primers were designed to perform ChIP-qPCR assay for each individual gene according to the results of COUP-TFII-ChIP-seq and YAP1-ChIP-seq analyses (Supplementary Fig. 5E–G). Results confirmed that COUP-TFII and YAP1 indeed bound to those gene loci (Fig. 5F). In summary, we demonstrate that COUP-TFII-YAP1 axis might be critical regulators to simultaneously regulate genes involved in several crucial processes for developing enzalutamide resistance.

### Therapeutic potential of YAP1 inhibition in vitro and in vivo

**Fig. 6:**
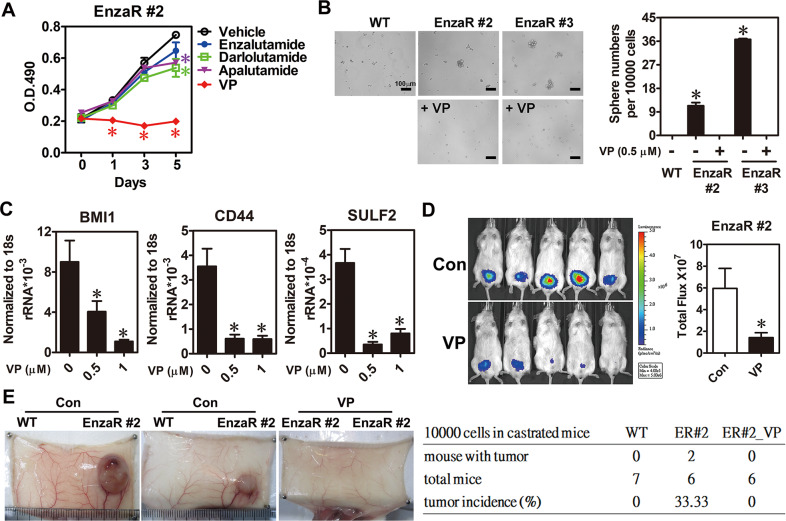
YAP1 inhibitor markedly attenuated growth and cancer stemness in enzalutamide-resistant cells. **A** EnzaR#2 cells were treated with 10 μM enzalutamide, darlolutamide, apalutamide or 0.5 μM Verteporfin (VP) for indicated time points and cell proliferation was analyzed by MTS assay (*n* = 3). Asterisk indicates *p* < 0.05 using two-way ANOVA test. **B** EnzaR cells treated with vehicle or VP compound were used to perform tumor sphere assay. Tumor sphere with diameter greater than 50 mm was counted (*n* = 3). Representative pictures were shown in the left panel. Asterisk indicates *p* < 0.05 using two-tailed Student’s *t*-test. **C** Expression levels of cancer stemness markers were analyzed by using RT-qPCR in enzalutamide-resistant cells treated with different doses of VP compound for 24 h (*n* = 3). Asterisk indicates *p* < 0.05 using one-way ANOVA test. **D** EnzaR (#2) cells with stable luciferase were performed orthotopic injection into the mouse prostate of NOD-SCID mice with castration. Mice were intraperitoneally received 5% DMSO (Con) or VP (25 mg/kg) compound treatment twice a week for 1 month. Tumor growth was analyzed by IVIS spectrum imaging system and result was quantified in the right panel (*n* = 5 for each group). **E** LNCaP (WT) or EnzaR cells were pretreated with vehicle or VP (0.5 μM) for 24 h. Cells were individually trypsinized and subcutaneously injected 1 × 10^4^ cells into castrated SCID mice for 4 months. A representative picture of tumor was presented when mice were sacrificed for analysis. Tumor incidence rate in different groups was calculated in the right panel.

Since overexpression of COUP-TFII and YAP1 play crucial roles in developing enzalutamide resistance, to block their functions may have therapeutic potential for the treatment with enzalutamide resistance. However, only YAP1 has the small molecular inhibitor, veterporfin (VP), which disrupts YAP1-TEAD interaction [26]. Later, EnzaR cells were treated with VP compound and other novel AR inhibitors such as darlolutamide and apalutamide to investigate their effects on cancer cell growth and stemness. Results show that VP compound treatment not only significantly retarded cell growth (Fig. 6A and Supplementary Fig. 6) but also reduced sphere numbers (Fig. 6B) while darlolutamide and apalutamide only had minor inhibitory effects on EnzaR cells (Fig. 6A). Furthermore, COUP-TFII and YAP1 co-regulated genes such as CD44, BMI1, and SULF2 were decreased by VP compound treatment in EnzaR cells (Fig. 6C). To further investigate the treatment effect of VP compound on tumor growth in vivo, EnzaR cells were orthotopically injected into male mouse prostate and mice were simultaneously castrated to deprive endogenous androgen level. Then, VP compound (25 mg/kg) was given by intraperitoneal injection twice a week for 1 month. As shown in Fig. 6D, EnzaR cells still kept growing even without androgen in the control group of mice. However, VP compound treatment markedly inhibited enzalutamide-resistant tumor growth (Fig. 6D). Next, to evaluate VP compound effect on cancer stemness, LNCaP parental cells or EnzaR cells (1 × 10^4^) were pretreated with or without VP compound for 24 h and subcutaneously injected into castrated male mice. After inoculation for 4 months, 33.33% (2/6) of mice injected with EnzaR cells formed the solid tumors while mice injected with VP-pretreated EnzaR cells and LNCaP parental cells did not form any tumor (Fig. 6E). Taken together, these results suggest that VP compound has the therapeutic potential for enzalutamide-resistant patients.

### Diagnostic value and potential function of COUP-TFII/ YAP1 regulation axis in EVs isolated from enzalutamide-sensitive and -resistant specimens

**Fig. 7:**
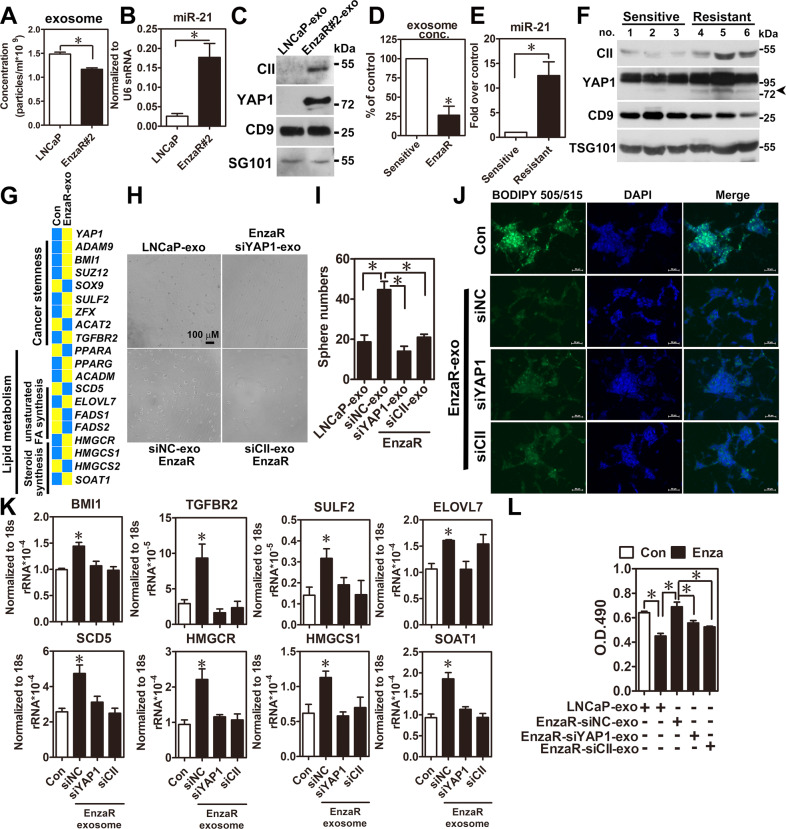
Increase of COUP-TFII/miR-21/YAP1 regulation axis in the EVs isolated from EnzaR cells and clinical specimens contributes to development of enzalutamide resistance. **A** Conditioned media from LNCaP parental and EnzaR cells were isolated EVs by size exclusion chromatography. Size and concentration of EVs were further analyzed by nanoparticle tracking analysis (NTA) (*n* = 3). **B**, **C** miR-21, COUP-TFII, and YAP1 expression levels were individually determined in EVs isolated from LNCaP parental and EnzaR cells by RT-qPCR (*n* = 3) and western blot. **D** Concentrations of EVs-isolated from sera of enzalutamide-sensitive and -resistant patients were determined by NTA analysis (*n* = 3). Results were presented as percentage of control. **E**, **F** miR-21, COUP-TFII, and YAP1 expression levels were individually determined in EVs isolated from sera of enzalutamide-sensitive and -resistant patients RT-qPCR (**E**) (*n* = 3) and western blot (**F**). **G** Heatmap showed the expression profiles of genes related to cancer stemness and lipid metabolism in LNCaP cells treated with EnzaR-EVs (10 μg) for 48 h. **H**, **I** LNCaP cells were treated with control (siNC), YAP1-, or CII-depleted EnzaR-EVs (10 μg) for 12 days for sphere culture (*n* = 3). Representative pictures and quantifications of sphere numbers were respectively shown in the **H**, **I**. **J** LNCaP cells treated with EnZaR-EVs derived from siNC, siYAP1, and siCII in EnzaR cells for 48 h were performed lipid staining by BODIPY 505/515 dye. **K** LNCaP cells were treated with control (siNC), YAP1-, or CII-depleted EnzaR-EVs (10 μg) for 48 h. Expression levels of cancer stemness-related genes such as BMI1, CDK6, TGFBR2, and SULF2 were determined by RT-qPCR (*n* = 3). **L** LNCaP cells were co-treated with enzalutamide (10 μM) and LNCaP-EVs or control (siNC), YAP1-, or CII-depleted EnzaR-EVs (10 μg) for 72 h to perform cell proliferation assays (*n* = 3). Asterisk indicates *p* < 0.05 using two-tailed Student’s *t*-test for above experiments.

In clinical, it is challenging to get the tissue specimens from the enzalutamide-resistant patients because clinical doctors do not recommend surgery as a treatment option at this stage. However, serum of patient is routinely drawn for check of prostate-specific antigen (PSA) level after starting enzalutamide treatment. Therefore, we ask whether COUP-TFII-miR-21-YAP1 regulation axis has diagnostic value for predicting enzalutamide resistance. First, COUP-TFII, miR-21, and YAP1 were found in Vesiclepedia, a database collecting contents of extracellular vesicles (EVs), suggesting they can be secreted into the extracellular microenvironment. To test the idea, conditioned media from EnzaR and LNCaP parental cells were used to isolate EVs by using size exclusion chromatography. Nanoparticle tracking analysis (NTA) further analyzed size distribution and concentration of EVs from both cell types. Results revealed that the concentration of EVs from EnzaR cells was lower than LNCaP cells while size distribution from both cell types was no noticeable difference (Fig. 7A, Supplementary Fig. 7A, B). Next, COUP-TFII, miR-21, and YAP1 expression levels in EVs isolated from EnzaR and LNCaP parental cells were measured by western blot and realtime PCR methods. Results showed that COUP-TFII, miR-21, and YAP1 expression levels were markedly increased in EVs of EnzaR cells (Fig. 7B, C). Similar results were observed by using sera of enzalutamide-resistant patient (Fig. 7D–F).

Next, to investigate the role of EVs-released from EnzaR cells, LNCaP cells treated with vehicle control and EnzaR-EVs were sent to perform RNA-seq and GSEA analyses. Results showed that cancer stemness signature was also enriched and most of genes related to cancer stemness and lipid metabolism were increased in the LNcaP cells received EnzaR-EVs treatment (Supplementary Fig. 7C and Fig. 7G). Importantly, EnzaR-EVs treatment enhanced sphere formation (Fig. 7H, I) and reduced lipid content (Fig. 7J) while EnzaR-EVs-derived from YAP1 or COUP-TFII knockdown EnzaR cells completely (Fig. 7H, I) or partially (Fig. 7J) abolished those phenomena in LNCaP cells. Furthermore, EVs released from EnzaR cells significantly increased genes related with cancer stemness and lipid metabolism while these regulations also attenuated in EVs isolated from YAP1 or COUP-TFII depleted EnzaR cells (Fig. 7K). Finally, EnzaR-EVs were used to investigate their effects on sensitivity for enzalutamide treatment in LNCaP cells. Results showed that LNCaP cells received EnxaR-EVs decreased the sensitivity of enzalutamide while those effects were abolished when YAP1 or COUP-TFII was individually depleted in EnzaR EVs by treatment with siRNA in EnzaR cells before isolating EVs (Fig. 7L). Taken together, exosomal COUP-TFII/YAP1 regulation axis may have predictive potential and contribute to developing enzalutamide resistance via induction of cancer stemness and lipid metabolism.

## Discussion

Enzalutamide, a second-generation antiandrogen drug, is a novel therapy widely used for mCRPC patients in clinical since US FDA has approved it in 2013 [7]. Recently, its indication has extended several times to cover chemotherapy-naive mCRPC [8], nonmetastatic CRPC (nmCRPC) [9] and metastatic castration-sensitive (mCSPC) patients approved by US FDA in 2019. However, not all PCa patients respond to enzalutamide treatment. Primary resistance for enzalutamide was observed in AFFIRM and PREVAIL trials with an incidence rate of 46% (AFFIRM) and 22% (PREVAIL). Unfortunately, the remaining patients with initial responses are eventually acquired resistance for enzalutamide within 1 year [7, 27]. Furthermore, a recent large cohort study in Japan has demonstrated that the incidence rate of acquired resistance is over 50% in mCRPC patients [28]. Therefore, to investigate the potential factor or underlying mechanism causing enzalutamide resistance is an emergent and crucial issue for the therapy of PCa patients.

Herein, YAP1 is identified as a critical factor involved in the development of enzalutamide resistance (Fig.1). Although previous studies have revealed the critical roles of YAP1 in developing CRPC [19–21], our study is the first group to dissect the role of YAP1 in enzalutamide resistance. Moreover, COUP-TFII, a TF upregulated in EnzaR cells by our previous study [22], positively regulated YAP1 expression through transcriptional and post-transcriptional controls (Figs. 2, 3). Previously, Jiang et al. have reported that AR forms a complex with EZH2 and DNMT3a to inhibit YAP1 expression by DNA methylation in LNCaP cells [29]. However, detail information of the binding motif for that complex in YAP1 locus is not clearly described in their study. Our findings provide an alternative way to explain YAP1 overexpression via AR-indirect manner in EnzaR cells.

Our RNA-seq analyses revealed that YAP1 downstream target gene involved in cancer stemness and lipid metabolism. Indeed, a higher percentage of CSC population, cancer stemness, and lipid metabolism-related genes were increased in EnzaR cells (Fig. 4). Since alternation of lipid metabolism has been linked to enzalutamide resistance [30] and maintenance of cancer stemness [23], our findings indicate that YAP1 is the potential link between lipid metabolism and cancer stemness. In addition, increase of HMGCR, a YAP1 downstream target gene, has been shown to confer resistance to enzalutamide treatment [31], suggesting that alternation of lipid metabolism by YAP1 might also contribute to the development of enzalutamide resistance. Next, an earlier study has shown that interaction between AR and YAP1 positively regulates AR downstream gene expression levels and promotes the switch from androgen-dependent to castration-resistant growth in PCa [21]. Herein, we report for the first time that COUP-TFII is an alternative TF to interact with YAP1 for co-regulation of cancer stemness-related genes when AR function is inhibited by enzalutamide in EnzaR cells (Fig. 5). Furthermore, a recent study has shown that loss of CHD1 promotes global chromatin changes and contributes to enzalutamide resistance via increasing critical driver TF expression such as COUP-TFI [32]. Interestingly, COUP-TFII was also identified in their supplementary results without any further investigation [32]. Our results clearly support the essential role of COUP-TFII in the development of enzalutamide resistance via regulation of YAP1 function. Since specific COUP-TFII inhibitor has been recently identified and shown the therapeutic potential in PCa [33], it will be expected to test its efficacy for overcoming enzalutamide resistance in the future.

Besides enzalutamide, several novel AR inhibitors such as apalutamide and darolutamide are recently approved by US FDA for the treatment of patients with nmCRPC [34]. Among them, only darolutamide, an AR antagonist targeted both wild and mutated types of ARs, has been shown its potential therapeutic effect on enzalutamide resistance in vitro and in vivo [35]. Here, our findings indicated that both apalutamide and darolutamide had significant but with minor inhibitory effects on the growth of EnzaR cells. In contrast, VP compound, a YAP1 inhibitor, not only repressed the growth but also reduced cancer stemness of EnzaR cells in vitro and in vivo (Fig. 6). Obviously, the potential therapeutic effect of VP compound was better than those antiandrogen drugs in EnzaR cells (Fig. 6A). A recent study also has shown that IKBKE, a noncanonical I-kappa-B kinase, increases YAP1 expression via turnover of LATS2 expression by kinase-dependent ubiquitination and IKBKE inhibitor markedly repressed the growth of EnzaR cells [36]. Therefore, non-AR targeting strategy might be more effective to overcome enzalutamide resistance. In clinical, enzalutamide is always used for the treatment of CRPC patient as a single regimen [9]. Currently, a research has revealed that all the therapeutic strategies of enzalutamide cause the onset of resistance and combination therapies delay the onset of resistance to enzalutamide or eliminate the disease according to mathematical modeling with transgenic mouse model of PCa [37]. These results imply that combination therapies of enzalutamide and VP compound might prevent the development of enzalutamide resistance in CRPC patients.

When CRPC patients start to receive enzalutamide treatment, they will routinely be checked for PSA levels in the serum to monitor the efficacy of treatment and progression of the disease [28]. However, PSA level is occasionally elevated due to factors unrelated to disease progression such as use of herbal supplement and heterophilic antibody interference [38, 39]. Therefore, to identify a reliable biomarker for predicting enzalutamide resistance is an important issue in clinical. EV is a secreted vehicle ranging from 30 to 150 nm size with double membrane structure and it can embed different types of cargoes including DNA, RNA, and protein [40]. Due to EV provides relative stable microenvironment for its cargoes, to identify a specific biomarker for indicating development of the enzalutamide resistance from EV is a feasible way. However, only two EV-related studies have been investigated in enzalutamide resistance [41, 42] and only one study has demonstrated that AR-V7 is a predictive biomarker of resistance to hormonal therapy [41]. It is a pity that both studies do not investigate the pathological function of EVs secreted by EnzaR cells. Here, our results demonstrated that increases of COUP-TFII, YAP1, and miR-21 expression were detected in the EnzaR-EVs isolated from cells and sera of patients (Fig. 7A–F), suggesting their potential diagnostic values for the prediction of enzalutamide resistance. More importantly, treatment with EnzaR-EVs not only increased drug resistance but also cancer stemness and lipid metabolism abilities in LNCaP cells via the functions of COUP-TFII and YAP1 (Fig. 7G–L).

**Fig. 8:**
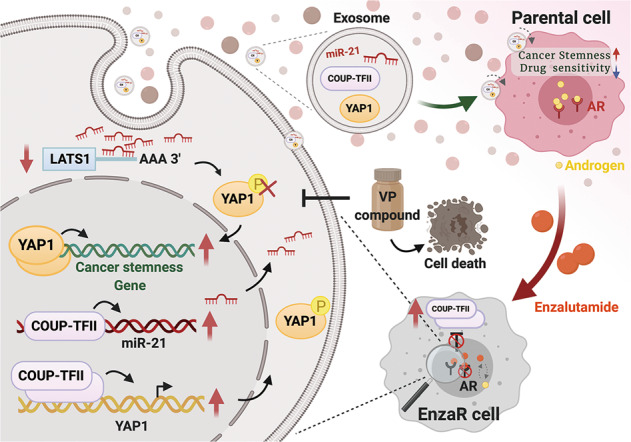
A cartoon briefly summarized the role of COUP-TFII/miR-21/YAP1 regulation axis in the development of enzalutamide resistance. Figure is created by BioRender.com.

In conclusion, we have revealed that overexpression of YAP1 plays crucial roles in the development of enzalutamide resistance and its underlying mechanism of overexpression is mediated by the function of COUP-TFII (Fig. 8). More crucially, YAP1 has the therapeutic and diagnostic potentials for enzalutamide resistance. Taken together, our findings shed light on new therapeutic strategy for enzalutamide resistance in the future.

## Materials and methods

Details of additional methodologies can be found in Supplementary Materials and methods.

### Clinical samples

Sera from enzalutamide-sensitive and -resistant patients were obtained from patients with PCa at the Department of Urology in the National Chung Kung University Hospital. Human Ethics Committee approval was obtained from the Clinical Research Ethics Committee at the National Cheng Kung University Medical Center, and each patient was signed for informed consent before collecting sample. Clinical information of patients were listed in the Supplementary Table 3

### RNA isolation, RT-qPCR, and western blot

Details procedures can be found in the Supplementary Materials and methods. Sequences of primers were listed in the Supplementary Table 4. Detail information of antibodies used in this study is shown in the Supplementary Table 5

### Animal models of PCa

Male NOD-SCID mice (8–10-week-old) were purchased from the Animal Center at the College of Medicine, National Cheng Kung University (NCKU). All the animal studies were approved by the Institutional Animal Care and Use Committee (IACUC:107242) in Laboratory Animal Center, NCKU. Detail experimental procedure for animal model of PCa can be found in Supplementary Materials and methods.

### Bioinformatics analysis

Details procedures can be found in the Supplementary Materials and methods. All the datasets used in this study were listed in the Supplementary Table 6.

### Statistical analysis

All data were presented as mean ± s.e.m. GraphPad Prism 5.01, a commercial statistical software, was used to perform all the statistical analysis. Statistical significance was set at *P* < 0.05 for all analyses.

## Supplementary information

Supplementary figures

Supplementary figure and table legends

Supplementary materials and methods

Supplementary table 1

Supplementary table 2

Supplementary table 3

Supplementary table 4

Supplementary table 5

Supplementary table 6
