# Supplementary material for: YAP1 overexpression contributes to the development of enzalutamide resistance by induction of cancer stemness and lipid metabolism in prostate cancer
Source: Oncogene. 2021 Mar 4;40(13):2407–21. doi: 10.1038/s41388-021-01718-4 (PMC8016667; doi:10.1038/s41388-021-01718-4)

**Supplementary figure 1**


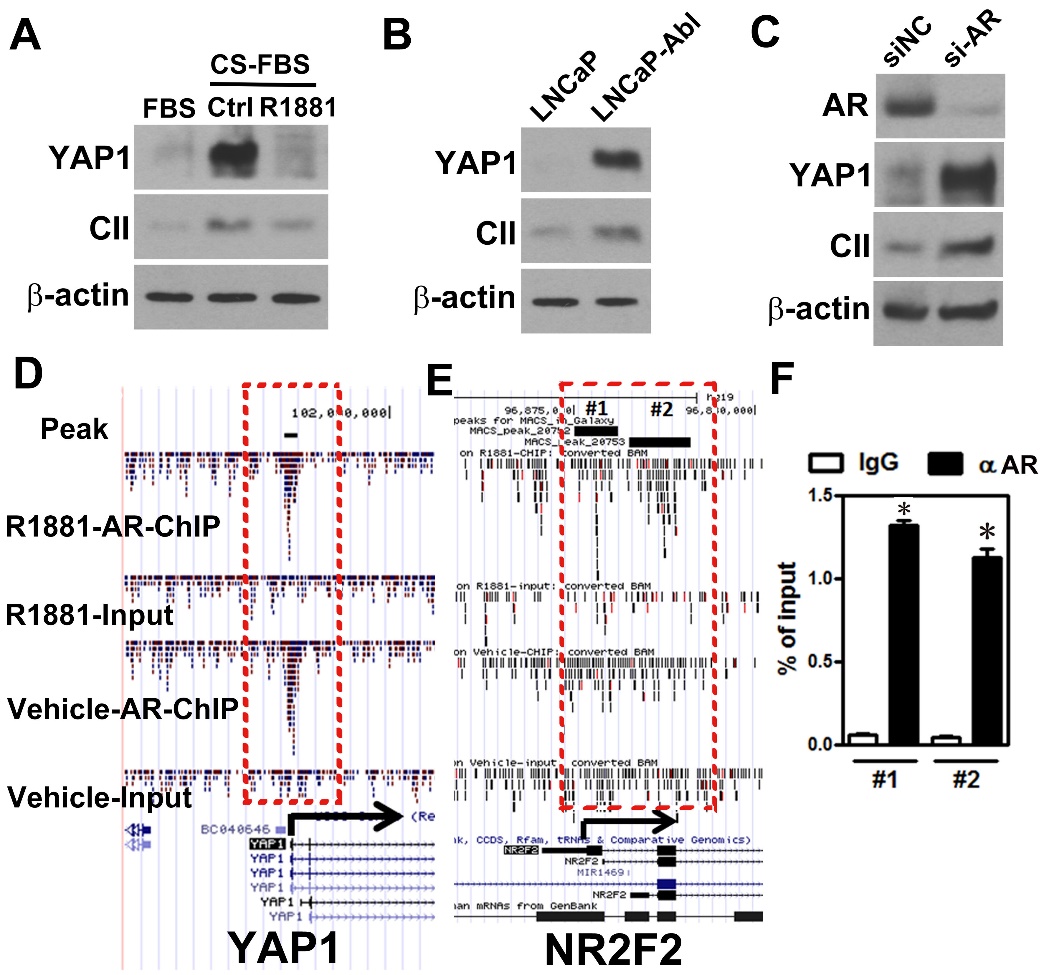


**
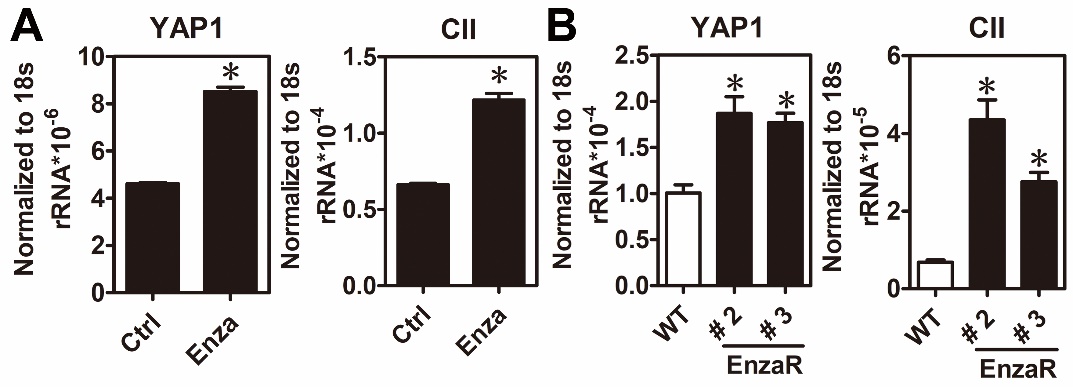
Supplementary figure 2:**

**Supplementary figure 3:**

**
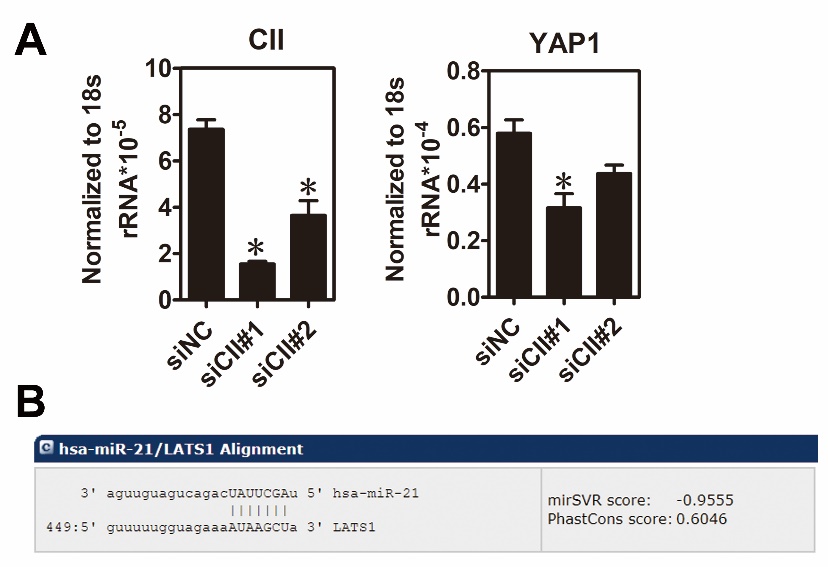
**

**
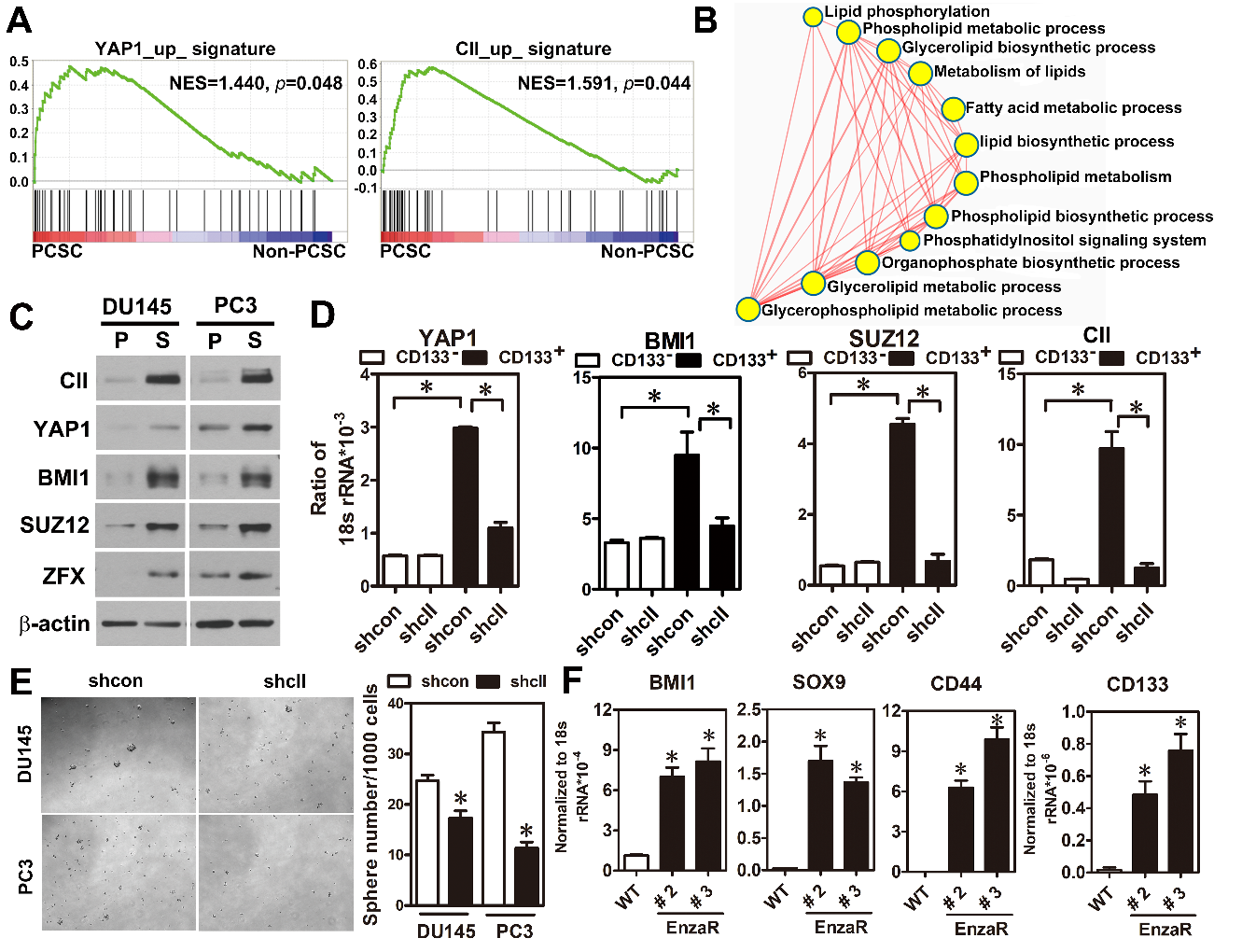
Supplementary figure 4:**

**Supplementary figure 5:**

**
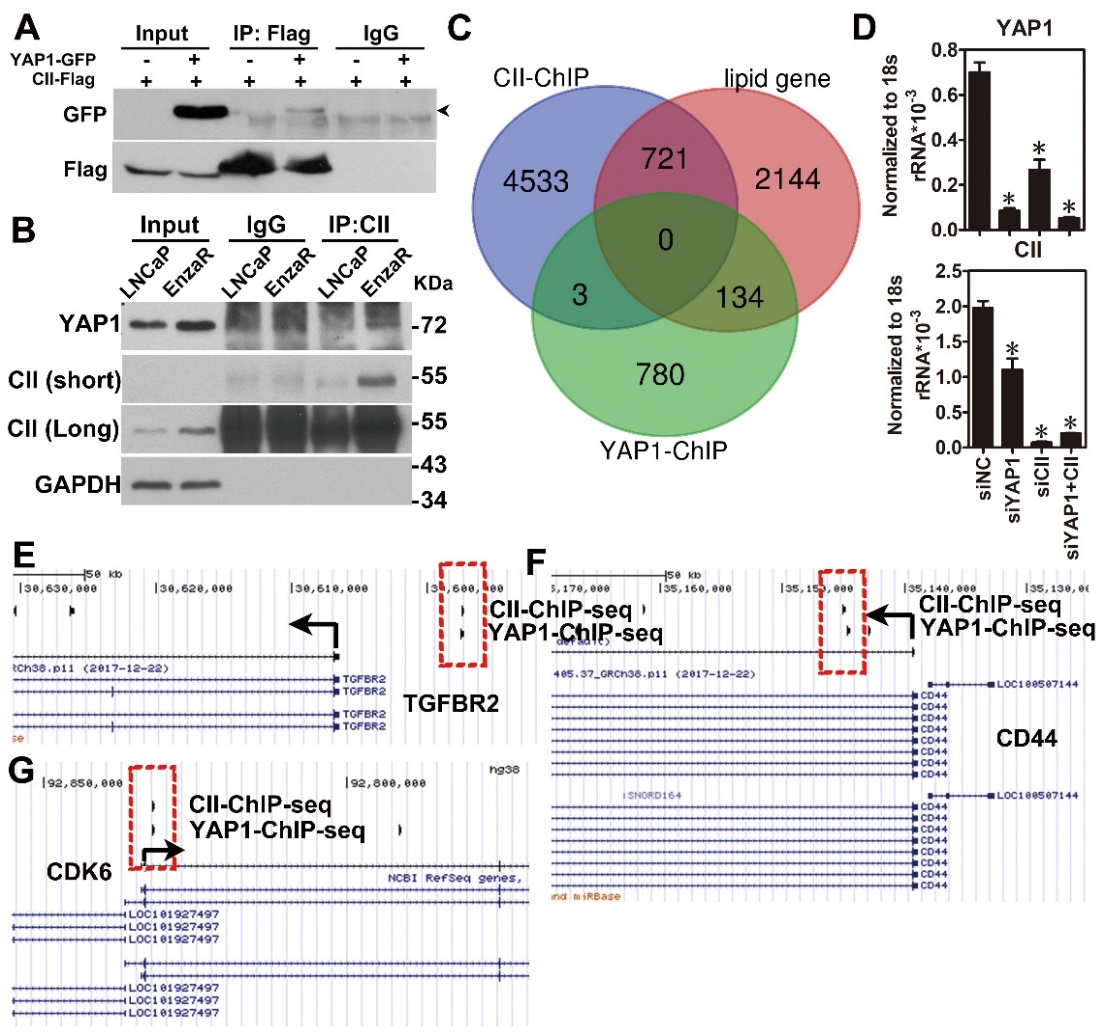
**

**Supplementary figure 6:**

**
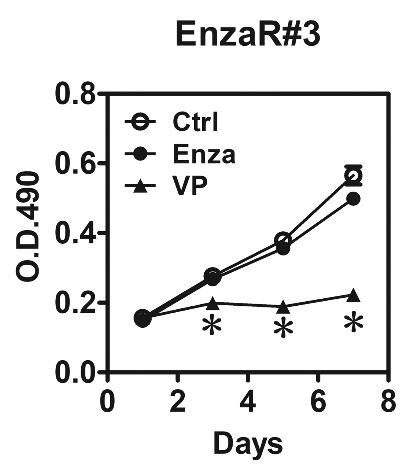
**

**Supplementary figure 7:**


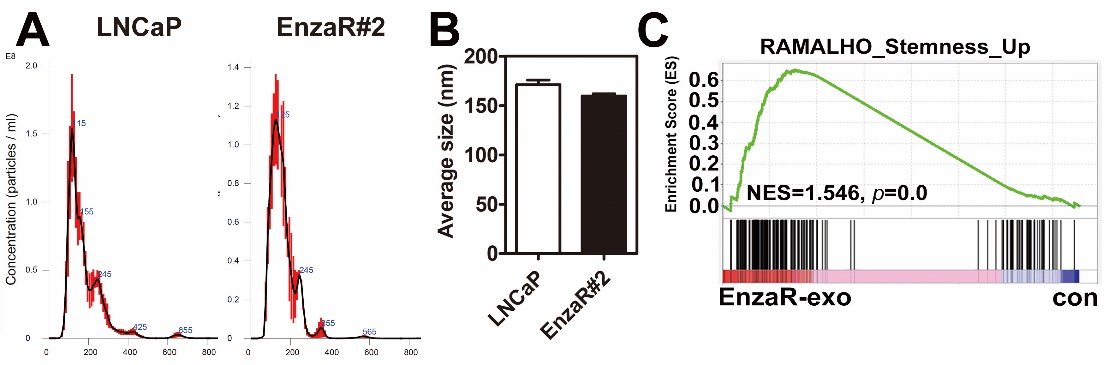

Supplement: Supplementary file 1 — Supplementary figures [file 41388_2021_1718_MOESM1_ESM.docx]
