# Supplementary material for: YAP1 overexpression contributes to the development of enzalutamide resistance by induction of cancer stemness and lipid metabolism in prostate cancer
Source: Oncogene. 2021 Mar 4;40(13):2407–21. doi: 10.1038/s41388-021-01718-4 (PMC8016667; doi:10.1038/s41388-021-01718-4)
Supplement: Supplementary file 2 — Supplementary figure and table legends [file 41388_2021_1718_MOESM2_ESM.docx]

**Supplementary figure 1: Androgen-AR regulation axis negatively regulates both COUP-TFII and YAP1 expression in LNCaP cells. (A)** LNCaP cells were treated with or without R1881 (10 nM) in the medium containing 10% charcoal stripped (CS)-FBS for 24 hours. YAP1, COUP-TFII (CII) and β-actin expression levels were determined by Western blot. **(B)** YAP1, COUP-TFII (CII) and β-actin expression levels were determined in LNCaP and LNCaP-ABl cell lines by using Western blot. **(C)** LNCaP cells were treated with control siRNA (siNC) or siRNA against AR (40 nM) for 48 hrs and detected YAP1, COUP-TFII (CII) and β-actin expression levels via Western blot. **(D, E)** a public dataset (GSE45124) performing AR-ChIP-seq by using LNCaP cells was downloaded and reanalyzed the raw data. AR binding signals in YAP1 **(D)** and COUP-TFII **(E)** loci were determined by peak-calling algorithm. **(F)** LNCaP cells were performed by AR-ChIP-qRT-PCR to verify bioinformatic findings in COUP-TFII locus. Primers (#1 and #2) were designed according to AR binding signals in COUP-TFII locus **(E)**.

**Supplementary figure 2: Both YAP1 and COUP-TFII expression levels are increased in enzalutamide-treated and -resistant cells. (A)** LNCaP cells were treated with enzalutamide (10 μM) for 72 hours. YAP1 and COUP-TFII (CII) expression levels were determined by qRT-PCR. **(B)** YAP1 and COUP-TFII (CII) expression levels were determined by qRT-PCR in LNCaP (WT) and two different enzalutamide-resistant (EnzaR) cells. Above results were normalized to an internal control gene, 18s rRNA.

**Supplementary figure 3: COUP-TFII-regulated miR-21/LATS1 axis is an additional regulation at the post-transcriptional level. (A)** EnzaR cells were treated with two different siRNAs against COUP-TFII (siCII) for 48 hours. COUP-TFII and YAP1 expression levels were determined by qRT-PCR. Results were normalized to 18s rRNA expression (n=3). **(B)** miR-21 targeting site located in 3’UTR region of LATS1 was predicted by microRNA.org software (<http://www.microrna.org/microrna/home.do>)

**Supplementary figure 4: COUP-TFII and YAP1 play crucial roles in cancer stemness and lipid metabolism in androgen-independent prostate cancer cell lines. (A)** YAP1 and COUP-TFII signatures were used to performed GSEA analysis by using with the cancer stemness dataset (E-MEXP-993)**. (B)** Downregulation of gene list (LogFC <-0.5, p<0.05) derived from YAP1 knockdown in EnzaR cells was analyzed by Metascape software (<http://metascape.org/gp/index.html#/main/step1>)**.** Genes involved in the lipid metabolism was shown as a enriched ontology cluster. **(C)** DU145 and PC3 were respectively cultured in the regular and ultralow dishes for 7 days. Expression levels of COUP-TFII (CII), YAP1 and cancer stemness-related genes inculding BMI1, SUZ12 and ZFX were assayed by Western blot. **(D)** PC3 cells carrying stable knockdown control (shcon) or shRNA against COUP-TFII (shCII) were sorted CD133^-^ and CD133^+^ cells by CD133 magnetic beads and extracted total RNA. COUP-TFII, YAP1, BMI1 and SUZ12 expression levels were measured by qRT-PCR. Results were normalized to 18s rRNA expression (n=3). **(E)** DU145 and PC3 cells carrying stable shcon or shCII construct were performed sphere culture for 7 days. Quantification of sphere numbers were counted from nine different areas of sphere images. **(F)**

**Supplementary figure 5: COUP-TFII and YAP1 co-regulated genes were identified by analyses of COUP-TFII- and YAP1-ChIP-seq data. (A)** Expression constructs of YAP1-GFP and COUP-TFII-Flag were individually or simultaneously transfected into 293FT cells for 24 hours. Flag antibody was used to perform immunoprecipitation assay and then GFP antibody was used to perform Western blot analysis. Black arrow indicated the signal of YAP1-GFP. **(B)** COUP-TFII antibody was used to pull down endogenous COUP-TFII protein and measured YAP1 expression in both LNCaP and EnzaR cells **(C)** Gene lists from YAP1-ChIP-seq (GSE66081) and COUP-TFII-ChIP-seq (GSE52008) were cross-referenced with genes related with lipid metabolism downloaded from GeneOntology to identify COUP-TFII and YAP1 co-regulated downstream targets that paly roles in lipid metabolism. **(E-G)** COUP-TFII and YAP1 ChIP-seq data were downloaded from ReMap2020 (<http://remap.univ-amu.fr/>). Next, those data were uploaded to UCSC genome browser. Red rectangles indicate gene containing both COUP-TFII and YAP1 binding signals in the same region.

**Supplementary figure 6: VP compound markedly inhibits the growth of EnzaR cells.** EnzaR (#3) cells were treated with enzalutamide (10 μM) or VP compound (0.5 μM) for indicated time points. Next, cell proliferation was analyzed by MTS assay (n=3).

**Supplementary figure 7: Analyses of EV characteristic and its potential function (A, B)** EVs were isolated from culture medium of LNCaP or EnzaR cells by using size exclusion column method. Next, EV sizes were no obvious difference by comparing those isolated from LNCaP and EnzaR cells after nanoparticle tracking analysis (NTA). **(C)** Gene expression profiles derived from LNCaP cells treated with EnzaR-exosome or vehicle control were performed GSEA analysis.

**Supplementary table 1: PCa patient information**

**Supplementary table 2: Primer list**

**Supplementary table 3: Antibody list**

**Supplementary table 4: Public datasets used in this study**

**Supplementary table 5**: GSE51873 datasets containing enzalutamide-sensitive and -resistant tumors were downloaded their expression values and analyzed oncogenic signatures by using GSEA analysis (Gene expression levels with significant overexpression over 0.5 fold in enzalutamide resistant tumors were used for analysis). YAP1 signature was enriched in the enzalutamide-resistant tumors.

**Supplementary table 6: Functional analyses of YAP1 and COUP-TFII co-regulated genes**
