# Supplementary material for: YAP1 overexpression contributes to the development of enzalutamide resistance by induction of cancer stemness and lipid metabolism in prostate cancer
Source: Oncogene. 2021 Mar 4;40(13):2407–21. doi: 10.1038/s41388-021-01718-4 (PMC8016667; doi:10.1038/s41388-021-01718-4)
Supplement: Supplementary file 3 — Supplementary materials and methods [file 41388_2021_1718_MOESM3_ESM.docx]

**Supplementary material and methods:**

**Clinical samples.** Sera from enzalutamide-sensitive and -resistant patients were obtained from patients with prostate cancer at the Department of Urology in the National Chung Kung University Hospital. Human Ethics Committee approval was obtained from the Clinical Research Ethics Committee at the National Cheng Kung University Medical Center, and each patient was signed for informed consent before collecting sample. Clinical information of patients were listed in the supplementary table 3

**Cell line.** LNCaP, EnzaR and PC3 cells were cultured in RPMI1640 medium with 10% FBS, antibiotics (100 μg/ml streptomycin and 100U/ml penicillin G), and 10 μM enzalutamide (only for EnzaR cells) in a humidified atmosphere of 5% CO_2_ and 95% air at 37℃. RWPE1 cells were cultured in keratinocyte serum free medium (K-SFM) with bovine pituitary extract (BPE, 0.05 mg/ml) and human recombinant epidermal growth factor (EGF, 5 ng/ml). Fresh mediums were routinely changed after two days of incubation and those cells were regularly checked for mycoplasma contamination by using a set of primer targeting 16 rRNA gene for PCR. Authentication of the cell lines were confirmed by the service of Center for Genomic Medicine in National Cheng Kung University.

**Drug treatment.** Darolutamide (Catalog No.S7559) and apalutamide (Catalog No.S2840) were purchased from Selleckchem.com (Houston, TX, USA). Verteporfin (SML0534), a YAP1 inhibitor, was purchased from Sigma-Aldrich (St. Louis, MO, USA). EnzaR cells were treated with 10 μM darolutamide, apalutamide, enzalutamide or 0.5μM verteporfin in RPMI1640 medium with 1% FBS for the indicated time points.

**RNA isolation and RT-qPCR.** Total RNA was isolated by TRIzol^TM^ reagent (Thermo Fisher Scientific, Waltham, MA USA) reagent according to the protocol provided by the manufacturer. 100 ng or 500 ng of total RNA was used to perform reverse transcription by TaqMan, MicroRNA Reverse Transcription Kit (Thermo Fisher Scientific, Waltham, MA USA) for miRNA or MMLV reverse transcriptase (Promega, Madison, WI, USA) for cDNA. Next, miRNA and mRNA transcripts were quantified by Applied Biosystems StepOnePlus real-time PCR machine (Thermo Fisher Scientific, Waltham, MA USA). 10 ng of miRNA transcript, 1X specific miRNA PCR primer and 10 μl 2X Taqman reagent (Thermo Fisher Scientific, Waltham, MA USA) were set up in one reaction for miRNA detection. For mRNA detection, 50 ng cDNA, 0.3 μM specific primer (Supplementary Table 4) and 10 μl SYBR Green mix (Roche) were used in each reaction.

**RNA-seq analysis.** Total RNA (2 μg) isolated from YAP1 knockdown in EnzaR cells and LNCaP cells treated with EnzaR-EVs were sent to performed RNA-seq analysis by AllBio science, Inc (Taichung, Taiwan). Original raw data and processed results had already submitted to the Gene Expression Omnibus (GEO) database (GSE164880).

**Western blot and Immunoprecipitation (IP).** Total cell lysates were isolated by RIPA buffer containing commercial protease inhibitors. 25 μg of protein was separated by SDS-polyacrylamide gel electrophoresis, and transferred to a polyvinylidene difluoride (PVDF) membrane. After blocking by 5% non-fat milk at room temperature for 1 hour, membrane was incubated by primary antibody at 4℃ overnight. Detail information of antibodies used in this study is shown in the supplementary table 5. To perform protein IP experiment, 2 μg of COUP-TFII-Flag and YAP1-GFP plasmids were transfected into 293T cells for 24 hours. Next, 500 μg of total cell lysates were added 1ug of Flag antibody (Merck & Co., Inc, Kenilworth, NJ, USA) or mouse IgG (Jackson ImmunoResearch, West Grove, PA, USA) to pull down exogenous COUP-TFII-flag protein. The interaction between exogenous COUP-TFII and YAP1 was analyzed by Western blot to detect Flag and GFP expression levels.

**siRNA and microRNA transfection.** 40 nM of siRNA (control, AR, COUP-TFII or YAP1), miR-21 mimic or inhibitor purchased from Thermo Fisher Scientific was transiently transfected into prostate cancer cells by Lipofetamine^TM^ 2000 (Thermo Fisher Scientific, Waltham, MA USA) for three days. Sequences of siRNA were listed in the supplementary table 4.

**Cell proliferation and migration assays.** EnzaR (2x10^3^) cells treated with verteporfin, darolutamide, apalutamide or 0.1% dimethyl sulfoxide (DMSO) as vehicle control were plated into 96 well plate. Next, cell proliferation assay was performed by the CellTiter 96® AQueous one solution cell proliferation reagent (Promega, Madison, WI, USA) according to the datasheet provided by manufacturer. Similar experiment was also performed in EnzaR cells pretreated with control siRNA and siYAP1 for 48 hours and then perform cell proliferation assay for indicated time points. EnzaR cells were knocked down by control siRNA or siYAP1 for 48 hours. Next, 2x10^4^ of EnzaR cells were plated into migration chamber with serum-free medium in the 24 well plate. Culture medium with 10% FBS was added in the well and then incubated for 24 hours. Then, cells were fixed by 4% paraformaldehyde and washed by 1X PBS. Migrated cells were stained by 0.25% crystal violet and counted from pictures taken at nine different areas in each experimental condition.

**Chromatin-immunoprecipitation (ChIP) assay.** ChIP assay was performed according to the manufacturer’s protocol by Pierce^TM^ Magnetic ChIP kit (Thermo Fisher Scientific, Waltham, MA USA). Briefly, cells were fixed by 1% formaldehyde and then fragmented by a combination of MNase treatment and sonication. Next, YAP1(D8H1X) (cell signaling, Cat#14074 ) or COUP-TFII (R&D system, Cat# PP-H7147-00) antibody was used to pull down YAP1-DNA complexes. Subsequently, DNA was isolated by washing, reverse crosslinking, treating with protease K (0.25 μg/ml) and collecting by purification column provided in the kit. The precipitated DNA was amplified by primers to verify the physical binding between YAP1/COUP-TFII and potential DNA region. Primer sequences were listed in the supplementary table 4.

**Ago2-RNA-immunoprecipitation (RIP) assay.** Ago2-RIP experiment was performed according to the manufacturer’s procedures by Pierce™ Magnetic RNA-Protein Pull-Down Kit (Thermo Fisher Scientific, Waltham, MA USA). 40 nM of microRNA inhibitor control and miR-21 inhibitor were individually transfected into EnzaR cells for 48 hours. Ago2-miR-21-mRNA complexes were pulled down by Ago2 antibody (Cat#RN003M) purchased from.MBL life science. RNA was extracted and analyzed by StepOnePlus real-time PCR machine. Primer information was listed in the supplementary table 4

**Sphere formation.** LNCaP, EnzaR, and EnzaR Cells (1x10^4^) pretreated with vehicle/verteporfin (0.5 μM) or knocked down by siRNA control, siYAP1 or siCOUP-TFII for 48 hours were cultured in ultra-low attachment 96 dish (CORNING, New York, USA ) by using serum-free DMEM/F12 medium containing recombinant human EGF (20 ng/mL), basic FGF (20 ng/mL), LIF (10 ng/mL), and insulin (5 μg/mL) for 7 to 12 days. Sphere number was counted by taking representative pictures from three different batches.

**Flow cytometry analysis.** Cells were trypsinized by 0.25% trypsine and neutralized by FBS. After 600 xg centrifugation for 10 minutes, cell pellets were suspended and washed by using filtered Flow buffer (1x PBS containing 2mM EDTA and 1% FBS). The cells were stained using PE-conjugated anti-CD133/2 (Clone 293C3, Miltenyi Biotec, Bergisch Gladbach, Germany) and APC-conjugated anti-CD44 (Miltenyi Biotec, Bergisch Gladbach, Germany) antibodies at 4℃ in dark. After 20 minutes incubation and subsequent washing, the cells were suspended in 400 µl Flow buffer. Cell suspension was immediately analyzed by using flow cytometry BD FACSCanto II and FlowJo software (Becton-Dickinson, San Jose, CA, USA).

**EV isolation and Nanoparticle Tracking Analysis.** Serum-free conditioned media from LNCaP and EnzaR cells or sera from enzalutamide-sensitive and resistant patients were centrifuged at 3000 g for 10 mins to remove cell debris. Next, size exclusion chromatography column (HansaBioMed, Tallinn, Estonia) was used to isolate EV from culture medium and serum according to the protocol provided by manufacturer. Subsequently, EV size and concertation isolated from culture medium and serum were calculated by NanoSight LM10-HS (Malvern Panalytical, Malvern, UK) in the center for Micro/Nano Science and Technology of National Cheng Kung University.

**Animal models of prostate cancer.** Male NOD-SCID mice (8~10-week-old) were purchased from the Animal Center at the College of Medicine, National Cheng Kung University (NCKU). All the animal studies were approved by the Institutional Animal Care and Use Committee (IACUC:107242) in Laboratory Animal Center, NCKU. No statistical methods were used for estimation of sample size in the animal study. Male mice were simultaneously castrated by surgery and performed orthotopic injection of EnzaR (2x10^6^) cells into mouse prostate for one month. After recovering for two weeks, mice were randomly separated (no blinding was done) into control group received vehicle control (5% DMSO) and VP group received verteporfin (25 mg/kg) twice a week for one month. Tumor growth was monitored by IVIS tracing system provided by the service of Laboratory Animal Center, NCKU. To set up cancer stemness model in vivo, male NOD-SCID mice were castrated by surgery and LNCaP or EnzaR (1x10^4^) cells pretreated with vehicle or verteporfin (0.5 μM) for 24 hours were subcutaneously inoculated for four months. Tumor pictures were taken when mice were sacrificed.

**Lipid staining.** LNCaP and EnzaR cells with different treatment conditions were used to perform lipid staining by BODIPY 505/515 (#D3921, Thermo Fisher Scientific, Waltham, MA USA) and results were analyzed by fluorescence microscopy.

**Bioinformatics analysis.** YAP1 and COUP-TFII signatures were respectively derived from Molecular Signatures Database of Gene Set Enrichment Analysis (GSEA) website and our previous data set GSE33182. Datasets of enzalutamide resistance (GSE52169) and prostate cancer stemness cell (E-MEXP-993) were individually downloaded from Gene Expression Omnibus (GEO) and GENOMESPACE databases to perform GSEA analysis by using YAP1 or COUP-TFII signature. Gene transcription signature correlation analyses of COUP-TFII and YAP1 in prostate cancer patients were described in previous study (1). COUP-TFII (GSE33182) and YAP1 (GSE7700, GSE32597, GSE35004 and GSE49406) signatures were analyzed in PCa patient data obtained from GSE21034 (Taylor), GSE10645 (Nakagawa) and TCGA PCa datasets. COUP-TFII physical binding sites located in both YAP1 and miR-21 loci were analyzed by ChIP-atlas tool (2). Finally, COUP-TFII-ChIP-seq (GSE52008), YAP1-ChIP-seq (GSE61852) and PCSC signature (GSE19713) were analyzed and cross-referenced to identify potential cancer stemness genes regulated by COUP-TFII and YAP1. All the datasets used in this study were listed in the supplementary table 6.

**Statistical analysis.** All data were presented as mean±s.e.m. A paired two-tailed Student’s t-test was used to compare differences between two groups and one-way AVNOVA followed by Dunnett post-analysis was used to compare differences more than two groups for RT-qPCR, migration, reporter and proliferation assays. Correlations of gene signatures were analyzed by Pearson’s correlation analysis. GraphPad Prism 5.01, a commercial statistical software, was used to perform all the statistical analysis. Statistical significance was set at P<0.05 for all analyses.

**Supplementary References:**

1. Lin SC, Kao CY, Lee HJ, Creighton CJ, Ittmann MM, Tsai SJ*, et al.* Dysregulation of miRNAs-COUP-TFII-FOXM1-CENPF axis contributes to the metastasis of prostate cancer. Nat Commun **2016**;7:11418 doi 10.1038/ncomms11418.

2. Oki S, Ohta T, Shioi G, Hatanaka H, Ogasawara O, Okuda Y*, et al.* ChIP-Atlas: a data-mining suite powered by full integration of public ChIP-seq data. EMBO Rep **2018**;19(12) doi 10.15252/embr.201846255.
